# A single-spheroid bovine mammary co-culture model for longitudinal monitoring and preclinical safety assessment of antimicrobial alternatives against mastitis-causing pathogens

**DOI:** 10.1186/s13567-026-01849-0

**Published:** 2026-11-04

**Authors:** Julie Bellemans, Yves Briers, Laurence Finot, Evelyne Meyer

**Affiliations:** 1 Laboratory of Biochemistry, Department of Veterinary and Biosciences, Faculty of Veterinary Medicine, Ghent University, Merelbeke, Belgium; 2 Laboratory of Applied Biotechnology, Department of Biotechnology, Faculty of Bioscience Engineering, Ghent University, Ghent, Belgium; 3 PEGASE, INRAE, Institut Agro Rennes-Angers, 35590 Saint-Gilles, France

**Keywords:** 3D in vitro model, mammosphere, BME-UV1, MAC-T, bovine mammary gland, casein, safety assessment, lysin, mastitis

## Abstract

Bovine mastitis remains a major driver of antimicrobial use in dairy production, highlighting the need for physiologically relevant in vitro models for preclinical safety assessment of novel intramammary therapeutics. Here, a previously established bovine epithelial cell line co-culture mammosphere model was adapted to a single-spheroid 96-well ultra-low attachment format, improving reproducibility and longitudinal monitoring. Mammospheres maintained epithelial organization and were further characterized for secretory function through assessment of casein expression. The suitability of the model for safety assessment was subsequently evaluated using complementary fluorescence microscopy, kinetic live-cell imaging, and flow cytometry following Triton X-100-induced cytotoxicity. Using this workflow, the safety of bacteriophage-derived recombinant lysin LysMK34 and antibiotic kanamycin, alone and in combination, was evaluated, with no cytotoxic effects observed, supporting the utility of the model for future preclinical intramammary safety assessment.

**Supplementary information:**

The online version contains supplementary material available at https://doi.org/10.1186/s13567-026-01849-0.

## Introduction, methods, and results

Bovine mastitis is one of the most prevalent and economically significant infectious diseases in the dairy industry, with a global incidence of subclinical and clinical mastitis of 42% and 15%, respectively [1]. In addition to its high prevalence, mastitis causes substantial economic losses due to reduced milk production, increased culling rates, treatment costs, and impaired animal welfare [2, 3]. Among the pathogens responsible for clinical mastitis, *Escherichia coli* is the most frequently isolated gram-negative causative agent, typically associated with acute and severe clinical signs [4, 5]. Its curative and preventive therapeutic management still relies typically on traditional systemic or intramammary antibiotics, making mastitis one of the leading drivers of antibiotic use in livestock production [6, 7]. Moreover, since January 2022, the European Union Veterinary Medicines Regulation 2019/6 has prohibited the routine prophylactic use of antibiotics in groups of animals. This highlights the increasing and currently urgent need for effective alternative antimicrobial strategies, preferably at the local level. In this context, bacteriophage-derived antimicrobials are gaining increasing interest either as a potential alternative to or an adjunct to conventional antibiotic therapies that are aimed at reducing antibiotic usage in veterinary medicine.

Lysins are bacteriophage-encoded peptidoglycan hydrolases that lyse infected bacterial cells at the end of the phage replication cycle. These enzymes act by targeting specific bonds within the peptidoglycan layer of the bacterial cell wall [8, 9]. Recombinant lysins can be used as enzyme-based antibiotics (“enzybiotics”) based on rapid peptidoglycan degradation and osmotic lysis upon their exogenous addition. These natural proteins have demonstrated strong bactericidal activity against gram-positive pathogens. However, their application against gram-negative bacteria has historically been limited by the presence of an additional outer membrane restricting access to the peptidoglycan layer [10, 11]. However, a limited subset of lysins, including LysMK34 derived from *Acinetobacter baumannii* phage PMK34, has been shown to possess intrinsic activity against gram-negative bacteria. Consequently, these lysins generally do not require outer membrane permeabilizers or prior genetic engineering to facilitate outer membrane penetration [12–15]. Although lysins are generally considered highly specific toward bacterial peptidoglycans, safety assessment on bovine mammary epithelial cells remains essential, particularly for lysins targeting gram-negative bacteria, which may contain membrane-active or amphipathic regions involved in outer membrane penetration [16]. However, before such novel antimicrobial agents can advance toward clinical application, their safety for the target host tissue (i.e., the bovine udder) must be assessed in vitro using bovine mammary epithelial cells (bMECs).

Such preclinical safety evaluation requires physiologically relevant in vitro models that recapitulate both the cellular architecture and functional properties of the secretory bovine mammary alveolus. Since mastitis predominantly occurs during early lactation, a physiologically relevant safety model should reproduce not only the epithelial organization of the mammary alveolus but also its secretory activity, including the capacity to produce milk proteins such as casein. Casein constitutes approximately 80% of total bovine milk protein and is a major marker of lactogenic differentiation in mammary epithelial cells [17–21]. Conventional two-dimensional (2D) cell culture models lack this structural complexity as well as the cell–cell interactions present in vivo, limiting their translational value [22–24]. In contrast, three-dimensional (3D) mammosphere systems allow bMECs to self-organize into multicellular aggregates that more closely mimic alveolar architecture and support epithelial polarization [25]. Our group recently established a novel matrix-free ultra-low attachment (ULA)-based bovine mammosphere model composed of co-cultured luminal BME-UV1 and basal MAC-T cells, providing a reproducible and animal-free platform supporting physiological epithelial organization, including tight junction formation [26]. As a major advantage compared with alternative extracellular matrix-embedded conditions, ULA provides a matrix-free environment, ensuring direct contact between the spheroid and any compound added at a defined concentration, which is particularly advantageous for safety screening applications [26, 27]. However, this ULA-based mammosphere system was established in 24-well plates, yielding large numbers of spheroids per well with variable size, thereby limiting reproducibility and longitudinal monitoring of individual spheroids. Yet, both latter criteria are essential for its use as a preclinical safety assessment tool and were therefore further explored in the current follow-up study.

The present study first aimed to improve the reproducibility of the model by establishing a single-spheroid setup in 96-well ULA plates and reassessing epithelial barrier formation and cell polarization through Zonula occludens-1 (ZO-1) and actin staining, respectively. Subsequently, its secretory competence was evaluated by assessing casein production. Next, the suitability of the model as an in vitro safety screening tool was investigated using complementary viability assessment approaches, including fluorescence microscopy, kinetic live-cell imaging and flow cytometry, following Triton X-100-induced cytotoxicity. Finally, these approaches were applied to assess the safety of the traditional aminoglycoside antibiotic kanamycin, used as a representative conventional intramammary antibiotic against gram-negative mastitis, and the bacteriophage-derived lysin LysMK34, selected as a proof-of-concept enzyme-based antimicrobial due to its previously reported intrinsic activity against gram-negative bacteria. The safety of both treatments, alone and in combination, was evaluated in the optimized co-cultured single-mammosphere model.

Co-cultured BME-UV1 and MAC-T mammospheres were reproduced in ULA conditions according to Bellemans et al. [26]. To generate a single mammosphere per well, two densities of BME-UV1 cells (2500 or 4000 cells in 200 µL of BME-UV1 proliferation medium per well of 0.32 cm^2^ surface area) were seeded in a U-bottom 96-well ULA plate. After seeding, the plate was centrifuged for 10 min at 250 *g* to cluster cells at the bottom of the wells. BME-UV1 cells were cultured as mammospheres for 7 days, including 5 days in BME-UV1 proliferation medium followed by 2 days in BME-UV1 differentiation medium. On day 7, 2500 MAC-T cells were added. The plate was again centrifuged for 10 min at 250 *g* to ensure cell contact with the mammosphere and maintained for an additional 4 days in differentiation medium consisting of a 1:1 (v/v) mixture of BME-UV1 and MAC-T differentiation media as optimized in Bellemans et al. [26].

In total, co-cultured mammospheres were grown for up to 11 days. Individual mammosphere growth dynamics (mammosphere diameter and area) were determined kinetically by phase-contrast imaging using a BioTek Cytation™ 5 Cell Imaging Multimode Reader integrated with a BioTek BioSpa Live-Cell Analysis System (Agilent Technologies, Santa Clara, CA, USA), enabling automated incubation and robotic plate transfer for longitudinal image acquisition. During mammosphere formation, images were acquired every 8 h from 16 h post-seeding at the initial spheroid formation until days 3 and 4, and then again from day 7 to day 11. Mammosphere diameter and surface area were quantified from the acquired images using Gen5 software version 3.12 (Agilent Technologies). Following the addition of MAC-T cells on day 7, the software was unable to distinguish between the BME-UV1 mammosphere and the individual MAC-T cells. Therefore, the first three time points, starting from day 7, were excluded from the analysis. Results were expressed as mean ± standard deviation (SD) and graphically presented using Excel (Microsoft).

For their immunofluorescence analysis, mammospheres were fixed in 4% paraformaldehyde (PFA) in phosphate-buffered saline (PBS) and immunostained following the procedure described by Bellemans et al. [26]. Primary antibodies targeting the luminal keratin (K)8, the basal K14, the tight junction protein ZO-1, and the cytoskeletal actin were used as detailed by Bellemans et al. [26]. All steps were performed under a binocular stereomicroscope to minimize mammosphere loss. Fluorescent images were acquired with a confocal laser scanning microscope, operated with Zen software (CLSM, LSM880, Zeiss, Paris, France).

To assess milk casein expression, proteins from mammospheres were extracted, quantified, and analyzed by western blotting as described by Arevalo Turrubiarte et al. [25]. Briefly, proteins were extracted from either pelleted co-cultured mammospheres or separately cultured 2D monocultures of BME-UV1 or MAC-T cells, using 60 µL of the radioimmunoprecipitation assay (RIPA) extraction reagent (89901, Fisher Scientific, France). After vortexing, incubation on ice for 5 min, and centrifugation at 13 000 *g* for 10 min at 4 °C, the soluble protein fraction was collected and quantified using the bicinchoninic acid (BCA) assay kit (23227, Fisher Scientific) according to the manufacturer’s recommendations. For each sample, 15 µg of protein was resolved by sodium dodecyl sulfate–polyacrylamide gel electrophoresis (SDS-PAGE) in a NuPage 4–12% Bis-Tris gel (NP0322BOX, Fisher Scientific) and electrotransferred onto polyvinylidene difluoride (PVDF) membranes. Membranes were incubated overnight at 4 °C with primary anti-bovine casein (251309, Clinisciences, France) or anti-β-actin (A5441, Merck, France) antibodies, diluted 1:1000 and 1:5000, respectively. Then, membranes were incubated with the appropriate secondary antibodies, anti-rabbit IgG or anti-mouse IgG (P0399 and P0161, DAKO, Denmark), diluted 1:5000, for 2 h at room temperature. Immunoreactive proteins were detected using an enhanced chemiluminescence (ECL) substrate (32106, Fisher Scientific), and chemiluminescence signals were captured using the ImageQuant™ LAS4000 Imager system (Cytiva Life Sciences, France). β-actin was used as a loading control.

For viability testing, first, a positive control was generated by incubating 11-day-old mammospheres for 24 h with the cell-permeabilizing Triton X-100 (37238, SERVA Electrophoresis GmbH, Germany), at a final concentration of 0.001% diluted in PBS. Mammospheres incubated with PBS alone for 24 h served as a negative control. The comparison between both controls was performed to evaluate the viability assessment methods for detecting cytotoxic effects in the single co-cultured mammosphere model. Subsequently, these methods were applied to assess the safety of LysMK34 (2 µM) and kanamycin (20 µg/mL; T832.3, Carl Roth, Germany) after 24 h exposure to concentrations relevant for antibacterial activity, either as standalone treatments or in combination. The production of LysMK34 protein, including expression and purification steps, was performed as described by Abdelkader et al. [13] (Additional file 1).

Three complementary approaches were used to assess viability, each performed on a separate set of identically treated single mammospheres. The first approach was based on the addition of propidium iodide (PI; P4864, Sigma, MO, USA) and Hoechst 33342 (H1399, Invitrogen, CA, USA) directly to the culture medium at final concentrations of 0.5 µg/mL and 2.5 µg/mL, respectively, immediately after exposure of mammospheres to the respective control or antimicrobial treatment conditions. PI fluorescence of mammospheres was subsequently imaged every 2 h during the 24-h incubation period using the Cytation™ 5 Cell Imaging system, enabling comparative longitudinal assessment of cell death across treatment conditions. The PI signal intensity was quantified from the acquired images using the Gen5™ software as integrated fluorescence intensity per spheroid and normalized to the initial time point (*t* = 0) to account for baseline differences between mammospheres. The second approach consisted of adding both fluorochromes at their respective concentration directly to the culture medium of identically treated mammospheres after 24 h of treatment incubation, followed by endpoint fluorescence imaging. The third approach consisted of subjecting mammospheres to a live/dead viability assay after 24 h of incubation, using the Viobility™ 488/520 Fixable Dye Kit (130–135-318, Miltenyi Biotec, Germany) according to the supplier's instructions. After staining, mammospheres were gently washed and fixed in 4% PFA, blocked with 2% bovine serum albumin (BSA), and counterstained with Hoechst 33342 (final concentration of 5 µg/mL). The endpoint fluorescence imaging was performed using a THUNDER epifluorescence microscope (Leica, Germany).

In addition, the viability of mammospheres was also assessed quantitatively by flow cytometry following their dissociation into single-cell suspensions. First, viability from mammospheres was again compared between the positive (Triton X-100) and negative (PBS) controls, culturing them in 24-well plates. Then, the antimicrobial compounds were again compared with the negative control using a 96-well ULA plate format. Prior to dissociation, the differentiation medium was carefully removed, and mammospheres were washed with PBS. For the 24-well plate, the plate was tilted at 45°, allowing mammospheres to sediment by gravity, after which the medium or PBS (after the washing step) was carefully removed. For the 96-well plate setup, the plate was centrifuged at 200 *g* for 5 min to obtain mammospheres at the bottom of the U-shaped wells, allowing the removal of the culture medium or PBS. Mammospheres were then dissociated into single cells by trypsinization with 0.25% trypsin–ethylenediaminetetraacetic acid (EDTA; T4049, Sigma-Aldrich, MO, USA; 500 µL per well for 24-well plates; 200 µL per well for 96-well plates) for 15 min at 37 °C and 5% CO_2_, with manual resuspension by pipetting every 5 min. For single-cell suspensions from the 24-well plate, cells were transferred to 1.5 mL microcentrifuge tubes and centrifuged at 300 *g* for 10 min. Cell pellets were subsequently resuspended in 100 µL PBS and transferred to a new 96-well V-bottom plate. Single cells from the ULA 96-well plates were retained in the same plate, centrifuged at 450 *g* for 5 min, and the supernatants were removed by plate inversion. A dual staining solution of Annexin V-FITC (ab14082, Abcam, UK; 1:500 dilution) and PI (final concentration 1 µg/mL, from a 50 µg/mL stock in PBS) was prepared in an incubation buffer [10 mM 4-(2-hydroxyethyl)-1-piperazineethanesulfonic acid (HEPES), 140 mM NaCl, 5 mM CaCl_2_·2H_2_O, pH 7.4]. Single dissociated cells were stained with 100 µL of this dual staining solution and incubated for 10 min at room temperature in the dark, after which cells were resuspended in 80 µL of incubation buffer. To enable accurate volumetric quantification, BD TruCount™ beads (340334, BD Biosciences, CA, USA) were resuspended in PBS containing 1% BSA, and 20 µL of the resulting bead suspension was added per sample. Flow cytometric analysis was performed with the CytoFLEX 3-laser flow cytometer (Analis, Ghent, Belgium). Absolute cell numbers were calculated based on TruCount™ bead technology, taking the dilution factor into account. Cell populations were classified using a quadrant gating strategy: viable cells corresponded to the Annexin V^−^/PI^−^ population, early apoptotic cells to the Annexin V^+^/PI^−^ population and the late apoptotic/necrotic cells to the Annexin V^+^/PI^+^ population.

Statistical analyses were performed using GraphPad Prism software (GraphPad Software version 10.6.1, CA, USA). Data normality was assessed using the Shapiro–Wilk test where applicable, while the distribution of residuals from repeated-measures analyses was assessed visually using normal quantile–quantile (Q–Q) plots. Endpoint flow cytometry data comparing the negative and positive control groups were analyzed using an unpaired two-tailed Student’s *t*-test. Endpoint flow cytometry data across the antimicrobial treatment groups and the negative control were analyzed using one-way analysis of variance (ANOVA) followed by Bonferroni’s multiple comparisons test. Longitudinal data, including mammosphere diameter and area during model optimization, kinetic PI fluorescence intensity, and changes in mammosphere diameter and area following treatment, were analyzed using two-way repeated-measures ANOVA with Geisser–Greenhouse correction, followed by Bonferroni’s multiple comparisons test. Graphs representing mammosphere diameter, area, kinetic PI fluorescence intensity, and Δ mammosphere diameter and area were generated using Microsoft Excel, whereas flow cytometry graphs were generated using GraphPad Prism. Data are presented as mean ± standard deviation (SD). Differences were considered statistically significant at *p* < 0.05.

### Adaptation of our novel bovine mammosphere model to a single spheroid screening format

First, the optimal seeding density of BME-UV1 cells required to generate reproducible single spheroids in a ULA 96-well plate format was determined. In a preliminary experiment, a seeding density of 2500 cells (*n* = 9) was compared with that of 4000 cells (*n* = 11). After 5 days of culture in proliferation medium, both conditions resulted in the formation of single-proliferating spheroids. However, spheroids generated from 4000 cells exhibited a significantly larger average diameter (368.65 ± 62.60 µm) than those generated from 2500 cells (290.74 ± 24.00 µm; *p* = 0.027), together with increased variability. In line with this, the average spheroid area was significantly higher in the higher seeding density condition (10.74 × 10^4^ ± 3.82 × 10^4^ µm^2^) compared with the lower seeding density condition (6.55 × 10^4^ ± 1.00 × 10^4^ µm^2^; *p* = 0.0082), again with more variability observed in the former condition (Additional file 2). Based on these preliminary findings, all subsequent ULA 96-well plate experiments were performed with a seeding density of 2500 BME-UV1 cells.

**Figure 1:**
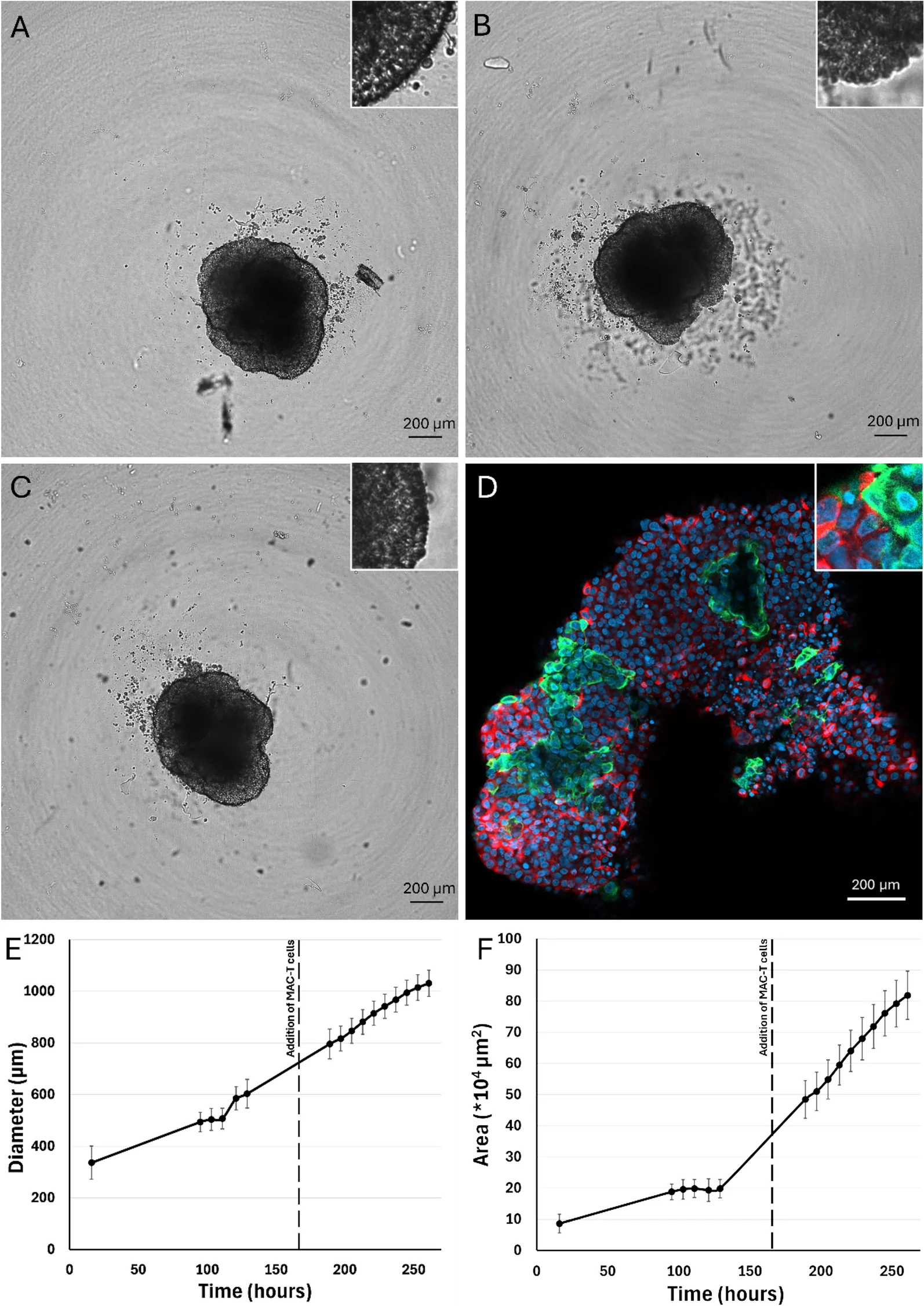
**Growth kinetics of single bovine mammospheres in a 96-well plate format**. Representative uncropped brightfield images acquired with live-cell imaging of three representative mammospheres formed after seeding 2500 BME-UV1 cells per well, showing typical spheroidal morphology at day 11 of co-culture (**A**–**C**). Fluorescence image of keratins (K) labeling in the co-cultured mammosphere with anti-K14 (green, MAC-T cells) and anti-K8 (red, BME-UV1 cells) antibodies and nuclei counterstained with Hoechst (blue), acquired by confocal laser scanning microscopy using Zen software (**D**). Mammosphere diameter (**E**) and area (**F**) over time from 16 h post-seeding to 11 days (261 h) of culture, demonstrating progressive growth during culture. Data are presented as mean ± standard deviation (*n* = 24). The dashed line indicates the time point of MAC-T cell addition.

After 11 days of culture, reproducible mammospheres (*n* = 24) displaying a consistent spheroidal morphology (Figure 1A–C), with a mean diameter of 1031.27 µm (±51.31 µm) and a mean surface area of 81.90 × 10^4^ µm^2^ (±7.73 × 10^4^ µm^2^), were obtained. Importantly, mammospheres consistently localized at the center of the U-bottom wells, enabling reliable automated and longitudinal monitoring using live-cell imaging. Occasional deviations from the typical spheroidal morphology were observed in some mammospheres (4/24; 16.7%; Additional file 3). These morphological deviations resulted from the progressive fusion of multiple smaller spheroids over time (Additional file 4). Immunofluorescence staining further confirmed the successful co-culture of luminal and basal bMEC, with K8-positive BME-UV1 cells (red) and K14-positive MAC-T cells (green) detected within the same mammosphere structure (Figure 1D). Overall, the 96-well ULA plate setup enabled the generation of single co-cultured mammospheres per well with acceptable reproducibility.

Kinetic monitoring of single mammospheres using a live-cell imaging system demonstrated a progressive increase in both their diameter and area over 11 days. Mammosphere diameter increased in a near-linear manner from 336.40 µm (±69.97 µm) at 16 h post-seeding to 1031.27 µm (±51.31 µm) at day 11 (261 h; Figure 1E), while the corresponding average surface area increased from 8.64 × 10^4^ µm^2^ (±3.01 × 10^4^ µm^2^) to 81.90 × 10^4^ µm^2^ (±7.73 × 10^4^ µm^2^) (Figure 1F). Notably, while the variability in diameter remained relatively stable over time, that of area increased, particularly after the addition of MAC-T cells.

### Structural and functional characterization of the single bovine mammospheres

**Figure 2:**
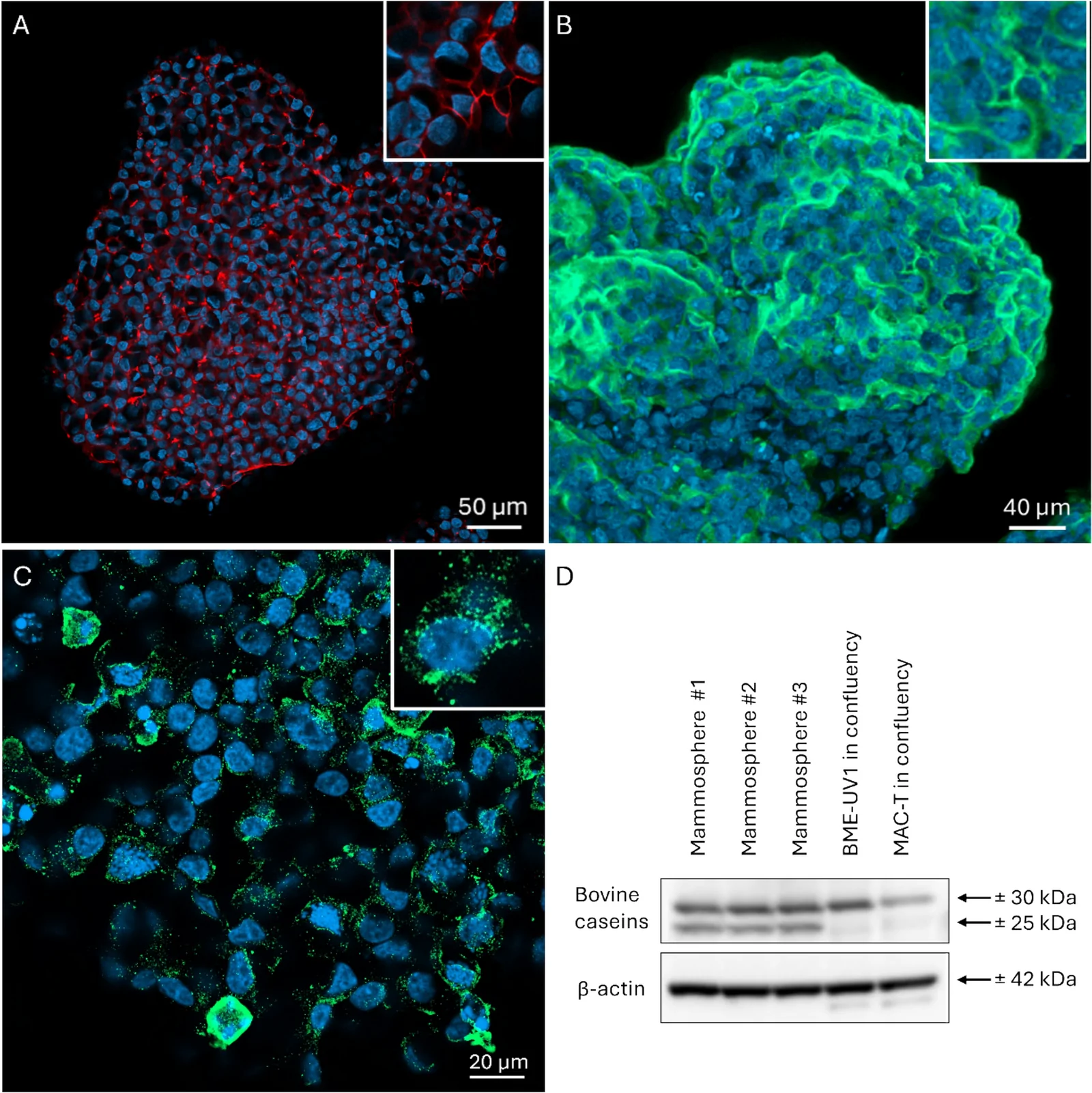
**Characterization of single bovine mammospheres**. Confocal microscopical images of Zonula occludens-1 (ZO-1; red; **A**) and actin (green; **B**) labeling illustrating epithelial barrier formation, and milk protein production capacity visualized by bovine casein labeling (green; **C**). Images were acquired by a confocal laser scanning microscope. A twofold magnification of the ZO-1, actin structures, and casein presence is shown in the top right insets. Western blot analysis showing casein protein expression in three independent mammosphere replicates compared with separately cultured 2D BME-UV1 and MAC-T cells (**D**). β-actin was used as a loading control. Protein size (expressed in kDa) is indicated.

To assess epithelial organization within the single co-cultured mammospheres, their tight junction expression and cytoskeletal organization were evaluated by confocal microscopy through Z-stack imaging (Figure 2A, B). A ZO-1 staining pattern consistent with Bellemans et al. was observed, characterized by a sharp and continuous localization at cell–cell borders, indicative of organized epithelial junctions (Figure 2A) [26]. Similarly, actin staining revealed a structured cytoskeletal network supporting the 3D mammosphere architecture (Figure 2B). These observations confirmed that comparable epithelial organization was reproducibly achieved in both 24-well (multiple spheroids per well) and 96-well (single spheroid per well) culture formats.

To further characterize the functional properties of our bovine mammosphere model, its secretory capacity was assessed by evaluating milk casein production. Confocal Z-stack imaging demonstrated the presence of casein throughout the mammospheres (Figure 2C). Complementary western blot analysis confirmed robust casein protein expression in mammospheres, whereas separately cultured 2D BME-UV1 and MAC-T cells showed lower casein expression and only a single casein band compared with the two bands observed in mammospheres (Figure 2D).

### Validation of viability assessment with complementary methods in single bovine mammospheres

To evaluate the suitability of the mammosphere model as a screening tool for candidate antimicrobial compound safety assessment, three complementary methods for viability assessment were validated. Cell viability was determined after 24 h treatment with 0.001% Triton X-100 as a positive control, compared with the sham-treated (i.e., PBS) negative control. Endpoint viability assessment was performed using two fluorescence imaging approaches, namely PI/Hoechst staining and Viobility™ dye/Hoechst staining, as well as quantitative Annexin V/PI flow cytometry. In parallel, kinetic fluorescence imaging using PI/Hoechst staining was applied for longitudinal monitoring of cell death during the 24 h treatment period.

**Figure 3:**
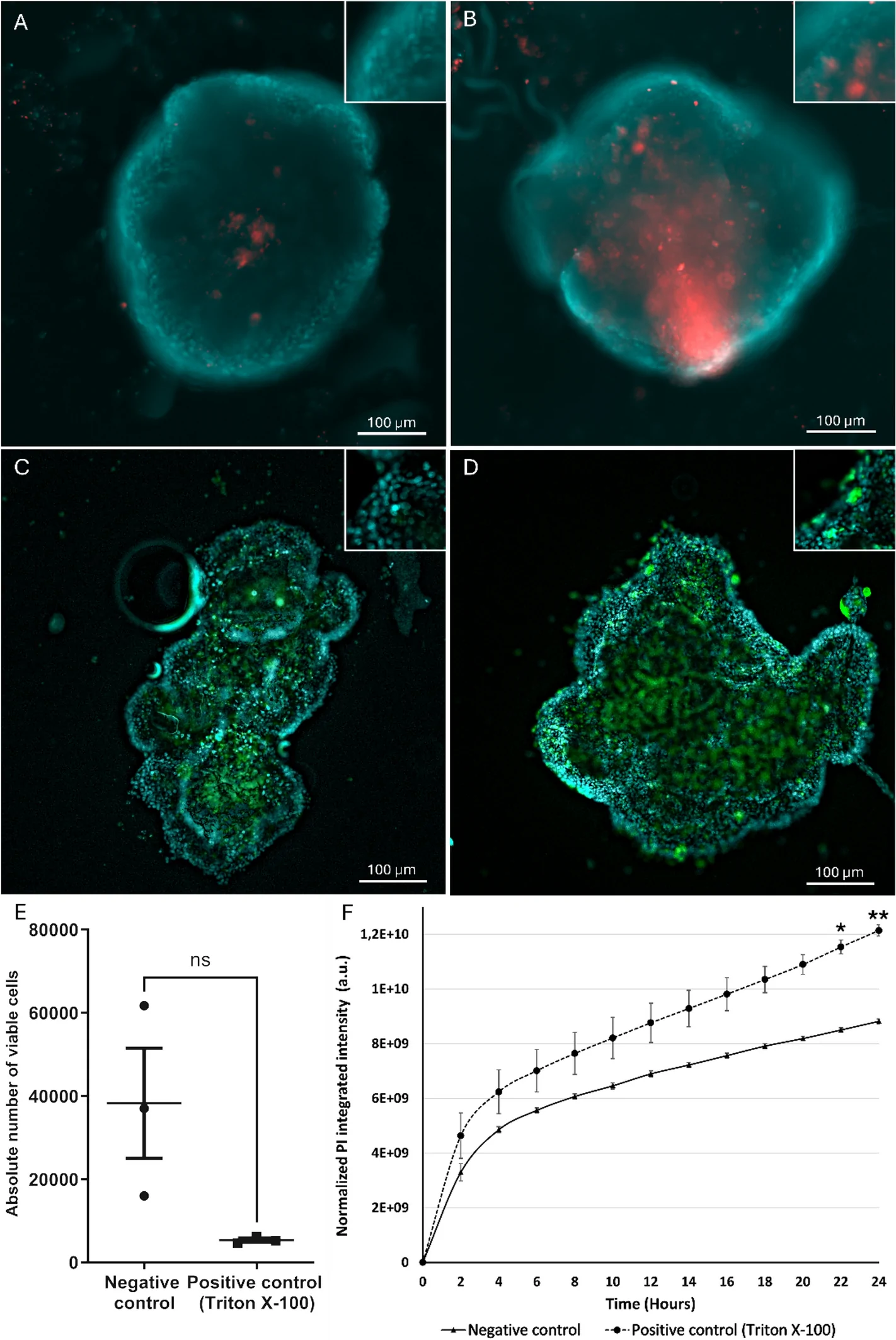
**Assessment of viability readouts in single bovine mammospheres following Triton X-100-induced cell death**. Endpoint fluorescence imaging of mammospheres incubated for 24 h with PBS (negative control) or 0.001% Triton X-100 (positive control). PI/Hoechst staining revealed PI-positive permeabilized cells (red)—albeit at a substantially different intensity—and nuclei stained with Hoechst (blue) in the negative (**A**) versus the positive (**B**) control. Live/dead fluorescence imaging using the Viobility™ dye (green) with Hoechst (blue) is shown for the negative (**C**) versus positive (**D**) control. Magnified insets (top right) highlight the mammosphere periphery to better visualize treatment-associated cell death. Images were acquired using fluorescence microscopy. Quantitative Annexin V/PI flow cytometric analysis of dissociated mammospheres showing the absolute number of viable cells in the negative versus positive control conditions (**E**). Kinetic live-cell fluorescence imaging using PI/Hoechst staining showing normalized PI integrated intensity over 24 h in negative versus positive controls (**F**). Images were acquired every 2 h using a live-cell imaging system. Data were analyzed using an unpaired two-tailed Student’s *t*-test (**E**) and a two-way repeated-measures ANOVA (**F**) and are presented as mean ± standard deviation (*n* = 3); ^*^p < 0.05; ^**^p < 0.01. Nonsignificance was considered *p* > 0.05. ns, nonsignificance.

Fluorescence endpoint imaging revealed the presence of either PI-positive cells or Viobility™ dye-labeled dead cells within the mammosphere core, indicating reduced viability in the mammosphere center (Figure 3A–D). In contrast, the outer cell layers remained largely viable in the negative control (PBS; Figure 3A, C), whereas substantial peripheral cell death was observed following Triton X-100 treatment (Figure 3B, D). Flow cytometric analysis of single dissociated cells showed a clear reduction in viable cell numbers following Triton X-100 treatment (5369 ± 814 cells) compared with the PBS control (38,245 ± 22,905 cells). However, due to the substantial variability within the negative control group, this difference did not reach statistical significance (*p* = 0.1; Figure 3E).

Kinetic fluorescence imaging using PI/Hoechst staining during 24 h showed PI accumulation progressively over time in both conditions, being consistently higher following Triton X-100 treatment compared with the negative control (Figure 3F). While differences were already apparent during the early incubation period, statistical significance was first reached only after 22 h of treatment, with PI intensity increasing from 8.50 × 10^9^ ± 8.35 × 10^7^ in the negative control to 1.15 × 10^10^ ± 2.56 × 10^8^ following Triton X-100 exposure (*p* = 0.013). This difference further increased over time, reaching 8.82 × 10^9^ ± 8.46 × 10^7^ and 1.21 × 10^10^ ± 2.06 × 10^8^, respectively, after 24 h (*p* = 0.0038). Longitudinal monitoring of changes in mammosphere diameter and area over the same incubation period revealed no significant differences between the positive and negative control conditions at any time point (Additional file 5).

### Safety assessment of the lysin LysMK34 and the aminoglycoside kanamycin, as standalone and combination treatments in single bovine mammospheres

To assess the safety of LysMK34, selected as a model antimicrobial compound of the novel class of enzybiotics, and kanamycin, single mammospheres were treated for 24 h with LysMK34 alone, kanamycin alone, or their combination and analyzed using the complementary viability assessment workflow.

**Figure 4:**
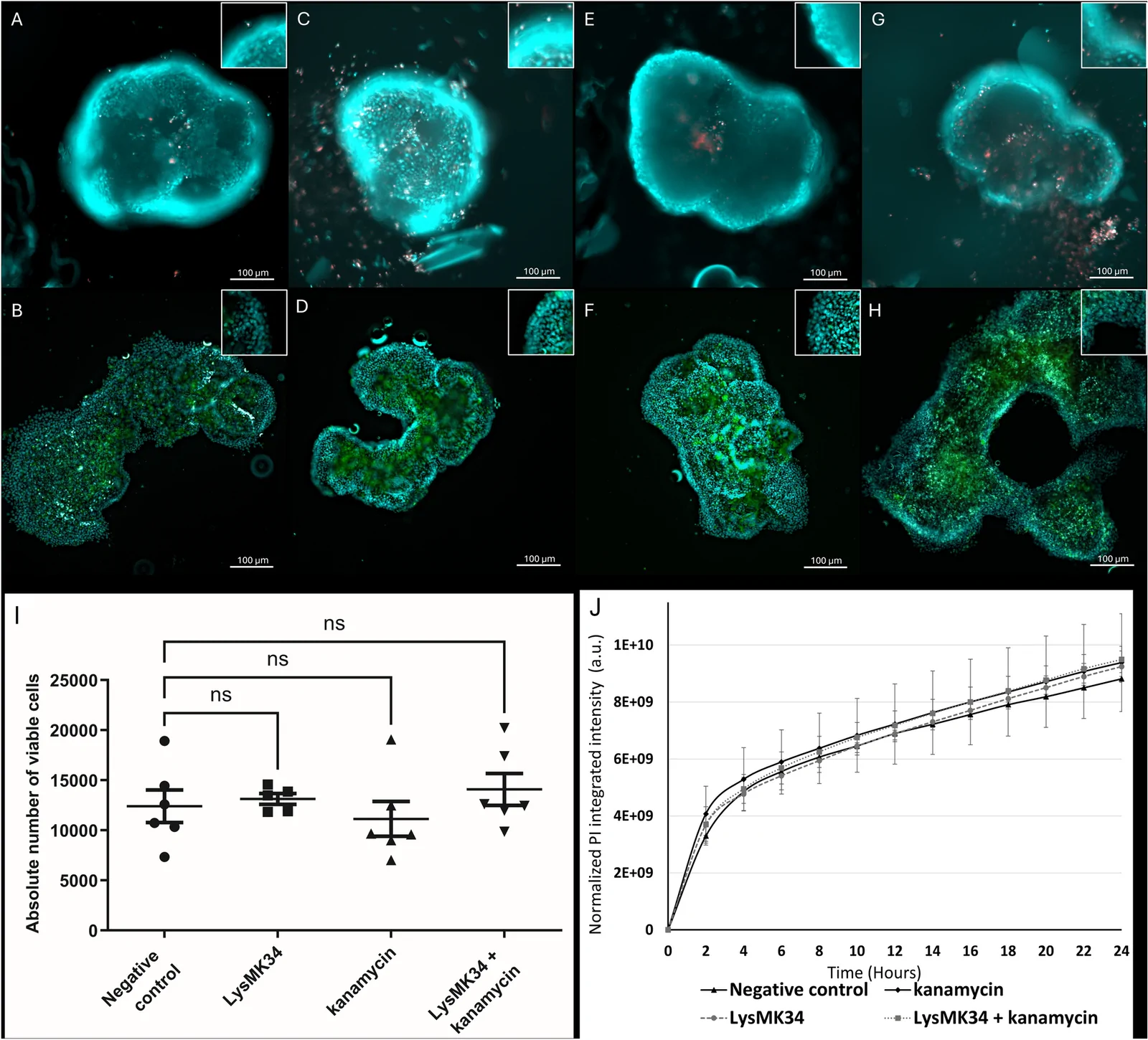
**Safety evaluation of LysMK34 and kanamycin, alone and in combination, in single bovine mammospheres**. Mammospheres were treated with LysMK34 alone, kanamycin alone, or their combination for 24 h. Endpoint fluorescence imaging of mammospheres treated with LysMK34 (**A** and **B**), kanamycin (**C** and **D**), combination of LysMK34 and kanamycin (**E** and **F**), or PBS as a negative control (**G** and **H**). Propidium iodide (PI; red) and Hoechst (blue) dual staining are shown in **A**, **C**, **E**, and **G**, whereas Viobility™ dye (green) and Hoechst (blue) dual staining are shown in **B**, **D**, **F**, and **H** using fluorescence microscopy. Magnified insets (top right) highlight the mammosphere periphery to better visualize staining at the outer cell layers. Viable cell numbers were quantified by flow cytometry (*n* = 6, except lysin-treated group *n* = 5) (**I**). Kinetic live-cell imaging of normalized PI integrated intensity was performed every 2 h (*n* = 3) (**J**). Data were analyzed using one-way ANOVA (**I**) and two-way repeated-measures ANOVA (**J**), and are presented as mean ± standard deviation; *p* > 0.05 is considered not significant. ns, no significant difference.

Endpoint fluorescence imaging revealed no increase in cell death at the mammosphere periphery following treatment with either LysMK34 alone (Figure 4A, B), kanamycin alone (Figure 4C, D), or their combination (Figure 4E, F) compared with the negative control (Figure 4G, H). A central accumulation of PI- and Viobility™-positive dead cells was observed at a comparable intensity across all conditions, indicating reduced viability in the spheroid core, as observed in the positive and negative control experiments. Flow cytometric analysis showed no significant differences in absolute viable cell numbers for LysMK34-, kanamycin-, or combination-treated mammospheres compared with the negative control, nor between treatment groups (Figure 4I), with average viable cell numbers of 13 137 ± 1207, 11 141 ± 4262, 14 086 ± 3892, and 12 399 ± 3983 cells, respectively. Similarly, kinetic PI fluorescence imaging over 24 h revealed no significant increase in cell death following exposure to LysMK34 alone, kanamycin alone, or their combination compared with the negative control, nor between treatment groups (Figure 4J).

## Discussion

This follow-up study aimed to adapt the previously established bovine co-cultured mammosphere model by Bellemans et al. toward a more reproducible single-spheroid screening tool suitable for longitudinal monitoring and preclinical safety assessment of novel intramammary therapeutics. For this purpose, the original matrix-free ULA 24-well co-culture system was adapted to a single-mammosphere 96-well format suitable for longitudinal monitoring. In parallel, it was assessed whether key structural characteristics previously observed in the original model [26], including epithelial organization and cytoskeletal organization, were maintained in this adapted configuration. In addition, the model was further functionally characterized through the assessment of milk casein production, and the suitability of complementary viability assessment methods was evaluated using Triton X-100 as a positive control. Finally, the applicability of the model for safety screening was investigated using the bacteriophage-derived lysin LysMK34 and the broad-spectrum antibiotic kanamycin, both as standalone treatment and in combination.

To establish a reproducible co-cultured mammosphere model, we focused on optimizing both single spheroid formation and cell viability monitoring. Our previously described ULA-based co-culture system successfully demonstrated that luminal BME-UV1 and basal MAC-T cells can coexist and self-organize into multicellular mammospheres displaying physiological epithelial characteristics [26]. This flat-bottom 24-well ULA setup enabled the generation of multiple mammospheres per well and provided a matrix-free environment, allowing for direct interactions between therapeutic compounds and the mammospheres. However, we also encountered several limitations. Due to the ULA conditions, mammospheres remained freely suspended, complicating the longitudinal monitoring of individual spheroids. In addition, substantial variability in spheroid size reduced overall experimental uniformity. Moreover, the corresponding mammosphere diameter variability was also high, i.e., approximately 35%. Finally, in the 24-well format, co-culture formation was not consistently achieved, as monocultured BME-UV1 spheroids were still observed [26]. In contrast, in our optimized 96-well single-mammosphere format, diameter variability was reduced to approximately 5%, while mammosphere area variability was approximately 9.4%, and co-culture formation was consistently achieved.

A critical parameter reported for reproducible spheroid formation is the initial cell seeding density. Although comparable studies have not yet been published for bMECs, several studies using cancer-derived 3D spheroid models have explored a wide range of seeding densities in 96-well formats. For example, Pereira et al. evaluated densities ranging from 2500 to as high as 20 000 cells per well, reporting that lower seeding densities (2500–5000 cells) are more suitable for continuously proliferating cell lines and longer culture durations [28]. Similarly, Vitacolonna et al. used seeding densities ranging from 500 to 5000 cells per well across different human cell lines, while Selby et al. reported optimal seeding densities of 1250–5000 cells per well, depending on the NCI60 cell line, resulting in spheroids with an average diameter of approximately 500 µm, as determined by imaging analysis [29, 30]. Considering the rapid doubling times of BME-UV1 of approximately 14 h and the extended culture duration envisaged for co-culture (> 10 days), a preliminary experiment was conducted comparing 2500 and 4000 seeded BME-UV1 cells. After 5 days in a proliferative condition, spheroids generated from 4000 BME-UV1 cells showed larger mean diameters and areas compared with those from 2500 cells. Moreover, increased variability in both parameters was also observed compared with the lower seeding density. Importantly, larger spheroids are prone to developing necrotic cores during prolonged culture as a result of hypoxia and diffusion-related limitations in the transport of nutrients and other molecules within the spheroid structure, as reported by us and others [26, 31]. Therefore, 2500 cells were selected as seeding density for subsequent characterization and safety assessment experiments. After 11 days of culture, this optimized setup yielded reproducible co-cultured mammospheres with regard to both their diameter and area. Still, some spheroids displayed minor morphological deviations, including irregular or multilobular spheroid structures. These deviations may result from the fusion of smaller cellular aggregates during spheroid maturation, a phenomenon that has been described in scaffold-free spheroid cultures and likely reflects ongoing dynamic cell–cell reorganization within the suspension environment [32]. We suggest that this should be taken into account when designing experiments, more specifically when determining the number of replicates required for screening applications. Nevertheless, this level of variability remained sufficiently limited to support further evaluation of the model for screening purposes. Both the single bovine mammosphere diameter and area increased over time, with diameter increasing linearly to approximately threefold between the proliferative phase and day 11 of co-culture differentiation. Notably, the area expansion followed a biphasic curve, becoming more pronounced following the addition of MAC-T cells and the switch to differentiation medium at day 7. Therefore, besides continued cellular proliferation, this nonlinear expansion may also reflect differentiation-associated cellular enlargement and increased secretory activity. Differentiated bMECs undergo substantial organelle remodeling, including expansion of the endoplasmic reticulum and Golgi apparatus required for milk protein synthesis and secretion [33]. In addition, the more pronounced variability observed for area may at least partly reflect differences in the extent of MAC-T cell attachment or distribution around single BME-UV1 mammospheres.

The introduction of centrifugation steps during both initial BME-UV1 cell seeding and subsequent MAC-T cell addition resulted in cellular aggregation and direct cell-spheroid contact at the bottom of the U-bottom wells. It promoted single and compact mammosphere formation, improving the efficiency and reproducibility compared with our original co-culture. This substantially improved spheroid uniformity, in turn, enabled reliable longitudinal tracking of single mammospheres using kinetic imaging.

Nevertheless, it was essential to confirm that key structural features of the original multispheroid model were preserved in the single-spheroids [26]. Co-cultured mammospheres generated in 96-well ULA plates expressed both ZO-1 and actin, indicating the formation of a primitive epithelial barrier and cytoskeletal organization. This demonstrates that the transition to a single-spheroid format does not compromise epithelial cell-to-cell integrity.

To further characterize the functional capacity of our model, its lactogenic differentiation was assessed through casein expression. Immunofluorescence microscopy showed intracellular casein localization, consistent with active synthesis and intracellular accumulation of these major milk proteins, as observed in secretory mammary epithelial cells in vivo. Complementary western blot analysis using a pan-casein antibody revealed two distinct casein bands in the 3D co-cultured mammosphere lysates, whereas only a single (and less intense) band was detected in 2D monocultures of both BME-UV1 and MAC-T cell lysates. Since all cultures were exposed to the same differentiation medium, the detection of an additional casein fraction in the 3D co-culture condition suggests that the more complex architecture and luminal–basal cell interactions may contribute to a broader activation of milk casein expression. These findings further support our earlier suggestion that the progressive increase in mammosphere diameter and area during differentiation may reflect not only proliferation but also lactogenic activity. Together, these observations support the physiological relevance of the model and its closer resemblance to the in vivo lactating mammary alveolus.

Based on these findings, we further investigated the applicability of the model as an in vitro platform for the initial safety assessment of candidate intramammary therapeutics, focusing on treatment-associated effects on mammosphere viability. Before evaluating the safety profile of LysMK34 and kanamycin, both as standalone treatments and in combination, we determined whether a cytotoxic effect was induced by the detergent Triton X-100 as a positive control. This effect was monitored with complementary viability assessment methods, including (i) endpoint fluorescence microscopy based on PI/Hoechst and Viobility™/Hoechst stainings to assess mammosphere viability and treatment-associated cell death, (ii) quantitative Annexin V/PI flow cytometric analysis to determine the number of viable cells, and (iii) kinetic live-cell fluorescence imaging using PI/Hoechst staining to monitor cell death in mammospheres over time.

Following Triton X-100 exposure, fluorescence imaging clearly demonstrated extensive peripheral cell death, whereas sham-treated (negative control) mammospheres largely retained viable outer cell layers. It should be remarked that cell death was observed in the mammosphere core in both conditions, which likely reflects the presence of diffusion-limited regions resulting in reduced oxygen and nutrient availability, as we previously described [26]. Flow cytometric analysis of dissociated mammospheres showed a clear reduction in viable cell numbers in the positive control compared with the negative control, but no statistically significant difference was observed. The latter was most likely caused by the substantial variability within the negative control group. Kinetic live-cell imaging revealed a progressive increase in PI intensity over time in both sham-treated and Triton X-100-treated mammospheres, likely reflecting, at least in part, the continued accumulation of PI-positive cells within the diffusion-limited mammosphere core described above. However, Triton X-100 treatment induced a significantly stronger increase in PI intensity, reaching approximately 1.4-fold higher levels after 24 h compared with sham-treated mammospheres. These findings confirm the ability of our methodology to detect treatment-associated cytotoxic effects. In contrast, longitudinal monitoring of mammosphere diameter and area did not reveal significant differences between Triton X-100-treated and sham-treated mammospheres.

Using this complementary viability assessment workflow, the safety profile of an in-house available lysin with activity against gram-negative pathogens (LysMK34) was subsequently evaluated. Kanamycin was included as a reference intramammary antibiotic also used against coliform mastitis and to assess the safety of potential combination therapy with LysMK34. Neither LysMK34 alone, kanamycin alone, nor their combination induced increased peripheral cell death compared with the sham-treated control. Similarly, no significant differences were observed in viable cell numbers or in PI intensity kinetics compared with the negative control. Together, these findings indicate that LysMK34, both alone and in combination with kanamycin, does not exert cytotoxic effects on the single-spheroid bovine mammary co-culture model under the tested conditions.

The absence of cytotoxicity following exposure to LysMK34 is particularly relevant given the increasing interest in bacteriophage-derived enzybiotics as alternatives or adjuncts to conventional intramammary antimicrobials for bovine mastitis [34]. The present findings, therefore, provide an important first indication that LysMK34 can be applied to bMEC co-cultures. Nevertheless, additional studies investigating longer exposure times, repeated administration, inflammatory responses, and ultimately in vivo safety and efficacy remain necessary before preclinical translation to in vivo (mouse and bovine) models can be considered.

As a future prospect, the physiological relevance of our model could be further enhanced. Indeed, ongoing advances in 3D culture engineering have demonstrated that epithelial cells can be grown around removable sacrificial scaffold templates to generate hollow spheroids with a centrally formed lumen. More specifically, Wang et al. (2017) showed that mouse lens epithelial cells can colonize gelatin microbead-templated spherical chambers, after which removal of the sacrificial microbeads resulted in hollow cell-lined spheroids [35]. Similarly, Lewis et al. (2015) earlier demonstrated that primary murine alveolar epithelial cells can be organized around photodegradable polyethylene glycol (PEG)-based microsphere templates, which, upon light-induced degradation, generated hollow epithelial cysts with a central lumen [36]. Applying such approaches to our bovine mammary co-culture model could support lumen formation more closely resembling the in vivo alveolar architecture, while also providing the spatial organization and apico-basal polarity required to further enhance milk casein expression and secretory function. Still, this remains to be established for the immortalized bMEC lines. Additionally, more complex co-culture with pre-adipocytes/adipocytes—which secrete paracrine factors that enhance mammosphere growth and, in combination with prolactin, stimulate αS1-casein synthesis in bMEC [37]—could provide an avenue to even further improve the physiological relevance of the current model. Nevertheless, it should be acknowledged that full recapitulation of the in vivo lactating mammary alveolus in vitro remains inherently limited by the absence of systemic hormonal cues, vascularization, and the complex stromal microenvironment present in intact and healthy udder tissue. In the infection context, the integration of (innate) immune cells, particularly neutrophils and potentially also macrophages, could represent an important next step toward a more pathophysiologically relevant in vitro bovine mammary gland model for (gram-negative) mastitis research. Studies have described methods for the recovery and characterization of bovine-milk-derived polymorphonuclear neutrophils and macrophages directly from raw milk, providing a physiologically relevant immune cell source that could potentially be incorporated into future co-culture systems [38–40]. Altogether, these refinements represent promising future directions toward a more comprehensive in vitro reconstruction of the lactating bovine mammary alveolus.

In conclusion, the present study further optimized the previously established bovine co-cultured mammosphere model into a reproducible single-spheroid platform suitable for longitudinal monitoring and preclinical safety assessment of candidate intramammary therapeutics. The adapted 96-well ULA configuration preserved key epithelial characteristics while additionally demonstrating secretory-related functionality through casein production. Using this model, the bacteriophage-derived lysin LysMK34, both alone and in combination with kanamycin, showed no cytotoxic effects under the tested conditions, supporting its further investigation as a potential antimicrobial candidate for intramammary applications.

## Supplementary information

**Additional file 1** **Protein expression and purification of LysMK34**. To produce recombinant LysMK34 protein, *E. coli* BL21(DE3) cells transformed with the pVTD9 expression plasmid encoding the LysMK34 gene and a kanamycin resistance cassette were pre-cultured for 18 h at 37 °C. This starter culture (5.0 mL) was subsequently transferred to a 2.0 L baffled Erlenmeyer flask containing 0.5 L lysogeny broth (LB) medium supplemented with kanamycin (50.0 µg/mL). Cultures were grown at 37 °C with shaking until an optical density (OD_600_) of 0.6–0.8 was reached, at which point recombinant protein expression was induced by adding isopropyl β-D-1-thiogalactopyranoside (IPTG; final concentration 1 mM). Following overnight expression at 16 °C for 18 h with shaking, bacterial cells were harvested by centrifugation (4000 *g*, 4 °C, 40 min). The supernatant was discarded, and the pellet was washed by resuspension in PBS pH 7.4, centrifuged again under the same conditions, and stored at −80 °C until further use. For cell lysis, the frozen pellet was thawed on melting ice and resuspended in lysis buffer (50 mM HEPES–NaOH, 500 mM NaCl, 10 mM imidazole, pH 7.4) supplemented with 1 mM phenylmethylsulfonyl fluoride (6367.1, Carl Roth, Germany) and 1 mM DNase I (M0303S, NEB, USA). Cells were disrupted by ultrasonication on melting ice (15 cycles of 15 s/10 s, amplitude 40%), followed by centrifugation (20 000 *g*, 4 °C, 20 min) to separate soluble from insoluble fractions. The supernatant (soluble fraction) was applied to a His GraviTrap™ column (GE11-0033-99, Sigma-Aldrich, USA) for immobilized metal affinity chromatography (IMAC). The column was first washed with wash buffer (50 mM HEPES–NaOH, 500 mM NaCl, 20 mM imidazole, 0.1% Empigen^®^, pH 7.4), followed by lysis buffer. Bound protein was eluted stepwise using increasing imidazole concentrations (50 mM and 500 mM) in elution buffer (50 mM HEPES–NaOH, 150 mM NaCl, 10% glycerol, pH 7.4). Eluted fractions were analyzed by SDS-PAGE on a 12.5% polyacrylamide gel, visualized by Coomassie Brilliant Blue G250 staining (9598.1, Carl Roth, Germany), and destained in distilled water. Fractions containing the target protein were pooled and subjected to buffer exchange into PBS pH 7.4 using Pierce™ Protein Concentrators PES with a 5 kDa molecular weight cutoff (88514, Thermo Fisher Scientific, USA), until the residual imidazole concentration was reduced to 0.5 mM. Protein concentration was determined by Bradford protein assay (5000205, Bio-Rad, USA).
**Additional file 2** **Growth dynamics of single bovine mammospheres using two different BME-UV1 cell seeding densities in a 96-well plate format over 5 days.** Growth kinetics of mammospheres cultured in proliferation medium over 5 days, assessed by longitudinal measurement of mammosphere diameter (A) and area (B), following seeding of 2500 BME-UV1 cells (*n* = 9) or 4000 BME-UV1 cells (*n* = 11). Data were analyzed using a two-way repeated-measures ANOVA and are presented as mean ± standard deviation; *p* values are represented as * and **, indicating *p* < 0.05 and *p* < 0.01, respectively.
**Additional file 3** **Representative examples of single bovine mammospheres displaying deviations from the typical spheroidal morphology are shown.** Representative uncropped phase-contrast images acquired with the Cytation™ 5 system showing two different single co-cultured bovine mammospheres display deviations from the typical spheroidal morphology after 11 days of culture in U-bottom ULA 96-well plates.
**Additional file 4** **Kinetic phase-contrast imaging of a representative mammosphere formed by co-cultured BME-UV1 and MAC-T cells at four time points, showing the formation of a single mammosphere from the clustering of multiple spheroids.** Representative kinetic phase-contrast images acquired using the Cytation™ 5 imaging system show the progressive formation of a single co-cultured mammosphere through the clustering and fusion of multiple smaller spheroids following seeding of 2500 BME-UV1 cells. Images were acquired at 5.5 days of BME-UV1 culture in proliferation medium (A), and following addition of MAC-T cells at 8.5 days (B), 10 days (C), and 11 days (D) in a 1:1 (v/v) mixture of BME-UV1 and MAC-T differentiation media. Progressive aggregation of multiple spheroids resulted in the formation of a single larger co-cultured mammosphere.
**Additional file 5 Longitudinal monitoring of mammosphere diameter and area following Triton X-100 treatment.** Longitudinal monitoring of mammosphere diameter (A) and area (B) following exposure to PBS (negative control) or 0.001% Triton X-100 (positive control). Mammospheres were imaged every 2 h from treatment onset until 24 h of incubation using phase-contrast imaging on the Cytation™ 5 Cell Imaging system. Mammosphere diameter and area were quantified using Gen5 software version 3.12. Changes over time were calculated relative to the initial time point (*t* = 0), and expressed as Δdiameter = diameter(*t*) − diameter(*t*_0_) and Δarea = area(*t*) − area(*t*_0_). Data were analyzed using a two-way repeated-measures ANOVA and are presented as mean ± SD (*n* = 3); ns indicates no significant difference (*p* > 0.05).

## Data Availability

The datasets generated and/or analyzed during the current study are available from the corresponding author upon reasonable request.
