# Supplementary material for: A single-spheroid bovine mammary co-culture model for longitudinal monitoring and preclinical safety assessment of antimicrobial alternatives against mastitis-causing pathogens
Source: Vet Res. 2026 Nov 4;57:194. doi: 10.1186/s13567-026-01849-0 (PMC13732830; doi:10.1186/s13567-026-01849-0)
Supplement: Supplementary file 1 [file 13567_2026_1849_MOESM1_ESM.zip › Bellemans et al. 2026_ Supplemental Files_Vet. Res..docx]

# Supplemental data

## Additional file 1. Protein expression and purification of LysMK34

To produce recombinant LysMK34 protein, *E. coli* BL21(DE3) cells transformed with the pVTD9 expression plasmid encoding the LysMK34 gene and a kanamycin resistance cassette were pre-cultured for 18 h at 37°C. This starter culture (5.0 mL) was subsequently transferred to a 2.0 L baffled Erlenmeyer flask containing 0.5 L LB medium supplemented with kanamycin (50.0 µg/mL). Cultures were grown at 37°C with shaking until an OD_600_ of 0.6-0.8 was reached, at which point recombinant protein expression was induced by adding IPTG (final concentration 1 mM). Following overnight expression at 16°C for 18 h with shaking, bacterial cells were harvested by centrifugation (4,000 g, 4°C, 40 min). The supernatant was discarded, and the pellet was washed by resuspension in PBS pH 7.4, centrifuged again under the same conditions, and stored at −80°C until further use. For cell lysis, the frozen pellet was thawed on melting ice and resuspended in lysis buffer (50 mM HEPES-NaOH, 500 mM NaCl, 10 mM imidazole, pH 7.4) supplemented with 1 mM phenylmethylsulfonyl fluoride (6367.1, Carl Roth, Germany) and 1 mM DNase I (M0303S, NEB, USA). Cells were disrupted by ultrasonication on melting ice (15 cycles of 15 s/10 s, amplitude 40 %), followed by centrifugation (20,000 g, 4°C, 20 min) to separate soluble from insoluble fractions. The supernatant (soluble fraction) was applied to a His GraviTrap™ column (GE11-0033-99, Sigma-Aldrich, USA) for immobilized metal affinity chromatography (IMAC). The column was first washed with wash buffer (50 mM HEPES-NaOH, 500 mM NaCl, 20 mM imidazole, 0.1% Empigen®, pH 7.4), followed by lysis buffer. Bound protein was eluted stepwise using increasing imidazole concentrations (50 mM and 500 mM) in elution buffer (50 mM HEPES-NaOH, 150 mM NaCl, 10% glycerol, pH 7.4). Eluted fractions were analyzed by SDS-PAGE on a 12.5% polyacrylamide gel, visualized by Coomassie Brilliant Blue G250 staining (9598.1, Carl Roth, Germany), and destained in distilled water. Fractions containing the target protein were pooled and subjected to buffer exchange into PBS pH 7.4 using Pierce™ Protein Concentrators PES with a 5 kDa molecular weight cut-off (88514, Thermo Fisher Scientific, USA), until the residual imidazole concentration was reduced to 0.5 mM. Protein concentration was determined by Bradford protein assay (5000205, Bio-Rad, USA).

**Figure: SDS-PAGE analysis of the overexpressed and purified recombinant LysMK34**. Lanes (L), from left to right, are as follows. Left Lane: PageRuler™ Unstained Broad Range Protein Ladder (26630, Thermo Fisher Scientific, USA), L1: insoluble fraction of the lysate of induced cells; L2: soluble fraction of the lysate of induced cells before metal affinity chromatography; L3: flow-through after metal affinity chromatography; L4: wash with wash buffer (20 mM imidazole with 0.1% Empigen®); L5: wash with lysis buffer (20 mM imidazole without Empigen®); L6: eluate (with 50 mM imidazole); L7 and L8: 1st and 2nd eluate of the purified LysMK34 (22.1 kDa) with 400 mM imidazole.

## Additional file 2. Growth dynamics of single bovine mammospheres using two different BME-UV1 cell seeding densities in a 96-well plate format over 5 days

Growth kinetics of mammospheres cultured in proliferation medium over 5 days, assessed by longitudinal measurement of mammosphere diameter **(A)** and area **(B)**, following seeding of 2,500 BME-UV1 cells (n = 9) or 4,000 BME-UV1 cells (n = 11). Data were analyzed using a two-way repeated-measures ANOVA and are presented as mean ± standard deviation; p values are represented as * and **, indicating p < 0.05 and p < 0.01, respectively.

## Additional file 3. Representative examples of single bovine mammospheres displaying deviations from the typical spheroidal morphology

Representative uncropped phase-contrast images acquired with the Cytation™ 5 system showing two different single co-cultured bovine mammospheres displaying deviations from the typical spheroidal morphology after 11 days of culture in U-bottom ULA 96-well plates.

## Additional file 4. Kinetic phase-contrast imaging of a representative mammosphere formed by co-cultured BME-UV1 and MAC-T cells at 4 time points, showing the formation of a single mammosphere from the clustering of multiple spheroids

Representative kinetic phase-contrast images acquired using the Cytation™ 5 imaging system showing the progressive formation of a single co-cultured mammosphere through the clustering and fusion of multiple smaller spheroids following seeding of 2,500 BME-UV1 cells. Images were acquired at 5.5 days (d) of BME-UV1 culture in proliferation medium **(A)**, and following addition of MAC-T cells at 8.5 d **(B)**, 10 d **(C)** and 11 d **(D)** in a 1:1 (v/v) mixture of BME-UV1 and MAC-T differentiation media. Progressive aggregation of multiple spheroids resulted in the formation of a single larger co-cultured mammosphere.

## Additional file 5. Longitudinal monitoring of mammosphere diameter and area following Triton X-100 treatment

Longitudinal monitoring of mammosphere diameter (A) and area (B) following exposure to PBS (negative control) or 0.001% Triton X-100 (positive control). Mammospheres were imaged every 2 h from treatment onset until 24 h of incubation using phase-contrast imaging on the Cytation™ 5 Cell Imaging system. Mammosphere diameter and area were quantified using Gen5 software version 3.12. Changes over time were calculated relative to the initial time point (t = 0) and expressed as Δdiameter = diameter_t_ − diameter_t0_ and Δarea = area_t_ − area_t0_. Data were analyzed using a two-way repeated-measures ANOVA and are presented as mean ± SD (n = 3); ns indicates no significant difference (p > 0.05).
